# Prognostic value of the nutritional risk index in patients with newly diagnosed multiple myeloma

**DOI:** 10.1007/s00277-022-05059-4

**Published:** 2022-11-28

**Authors:** Limei Zhang, Shuzhao Chen, Mayan Huang, Weida Wang, Yang Liang, Yun Wang

**Affiliations:** 1 State Key Laboratory of Oncology in South China, Collaborative Innovation Center for Cancer Medicine, Sun Yat-Sen University Cancer Center, Guangzhou, People’s Republic of China; 2 Department of Hematologic Oncology, State Key Laboratory of Oncology in South China, Sun Yat-Sen University Cancer Center, 651 Dongfeng Road East, Guangzhou, Guangdong 510060 People’s Republic of China; 3 Department of Pathology, State Key Laboratory of Oncology in South China, Sun Yat-Sen University Cancer Center, Guangzhou, People’s Republic of China

**Keywords:** Multiple myeloma, NRI, Survival, Prognostic factor

## Abstract

The nutritional risk index (NRI), which is based on weight and albumin levels, is closely associated with the prognosis of many cancers. However, its prognostic value has not been investigated in patients with newly diagnosed multiple myeloma (NDMM). We aimed to assess the association between the NRI and survival outcomes in patients with NDMM. We retrospectively collected and analyzed clinical and laboratory data from patients with NDMM between 2005 and 2019 at our center. Patients were stratified into the high NRI (> 89) and low NRI (≤ 89) groups for prognostic analysis. The NRI and other variables were also explored to evaluate their prognostic value for overall survival (OS). A total of 638 patients diagnosed with NDMM were retrospectively included. Patients in the high NRI group had a significantly better median OS than those in the low NRI group (64 months vs 43 months, *p* < 0.001). In the multivariate analysis, a high NRI was shown to be an independent prognostic factor for OS (hazard ratio, 0.758; 95% confidence interval, 0.587–0.977; *p* = 0.033). Age, performance status, transplant status, and lactate dehydrogenase level were also independent prognostic factors for OS. In conclusion, our study demonstrates that the NRI is a simple and useful predictor of survival outcomes in patients with NDMM.

## Introduction

Multiple myeloma (MM) is the second most common hematologic cancer in high-income countries and is characterized by the accumulation of monoclonal plasma cells in bone marrow and the production of immunoglobulin [1]. Because of the heterogeneity of MM, patient survival ranges from a few months to over 10 years []. Therefore, no single staging system can be used for all patients with MM, and several staging systems have been developed to predict prognosis and help risk stratification for patients with MM, including the Durie–Salmon (DS) staging system, international staging system (ISS), revised ISS (R-ISS), and Mayo Stratification of Myeloma and Risk-Adapted Therapy. All of these staging systems are based on two or three unfavorable prognostic variables [4,5,6,7]. Furthermore, an increasing number of cytogenetic and non-cytogenetic parameters have been explored to investigate their potential impact on MM prognosis.

In the era of novel drug development, the survival of patients with MM has been notably improved [8]. However, some patients with MM still do not obtain survival benefits from these drugs, and this phenomenon cannot be fully explained by biological and genetic prognostic factors [9, 10]. Treatment-related complications may explain the variability in overall survival (OS) in patients with MM.

Malnutrition is common in cancer patients, with an incidence ranging from 30 to 80% [11, 12]. Kim et al. reported that the malnutrition prevalence in MM was about 70% [13]. Some studies have found that malnutrition is highly associated with worse survival in cancer patients [13–15]. Further, malnutrition is related to the response to treatment and the occurrence of chemotherapy-related adverse events, which decrease survival [16–19]. The nutritional risk index (NRI), which is based on weight and albumin levels, is closely associated with prognosis in many cancers [20, 21]. However, its prognostic value for newly diagnosed MM (NDMM) has not been investigated.

Therefore, in this retrospective study, we aimed to investigate the association between the NRI and survival outcomes of NDMM patients.

## Patients and methods

### Patients

Patients diagnosed with NDMM between 2005 and 2019 at the Sun Yat-sen University Cancer Center (SYSUCC) were eligible for inclusion in this study. Baseline clinical and laboratory data were extracted from electronic medical records, including age, sex, disease stage, performance status, transplant status, weight, and β2-microglobulin (β2-MG), lactate dehydrogenase (LDH), C-reactive protein (CRP), creatinine (CRE), hemoglobin (HGB), albumin, and calcium (Ca) levels. The inclusion criteria were age older than 18 years and availability of complete baseline clinical and laboratory data (items noted above). Patients receiving only palliative treatment, those lost to follow-up, and those lacking baseline data were excluded from our study. The final follow-up was January 2022. Our study was approved by the institutional ethics committee of the SYSUCC. Our study was retrospective; therefore, the requirement for informed consent was waived.

### Statistical analyses

The NRI was calculated as follows: 1.489 × albumin (g/L) + 41.7 × (weight/usual body weight) [20, 21]. The cutoff value of the NRI was determined using maximally selected rank statistics; patients with an NRI > 89 were defined as the high NRI group, and the remaining patients were categorized into the low NRI group. Differences between the two groups were determined using the chi-square test or Fisher’s exact test. OS was defined as the interval between diagnosis and death due to any cause. The Kaplan–Meier method was used to estimate OS, and comparisons between groups were performed using the log-rank test. Univariate Cox regression analysis of OS was used to evaluate the prognostic value of the variables. Variables with *p* < 0.05 were included in a stepwise multivariate Cox regression analysis. A two-sided *p* value < 0.05 was regarded as significant. All statistical analyses were performed using R version 4.0.3 (The R Foundation, Vienna, Austria).

## Results

### Baseline characteristics

**Fig. 1:**
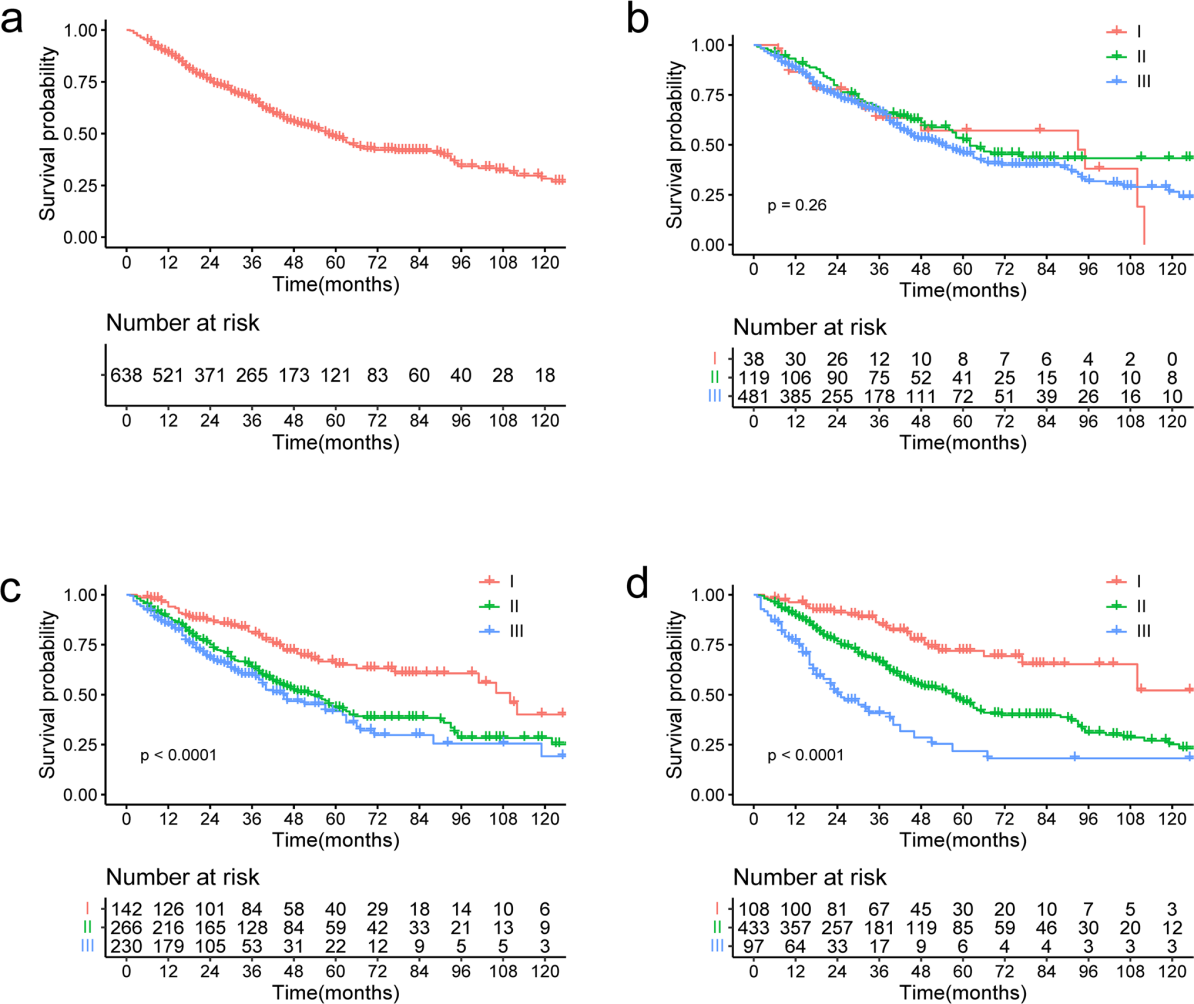
**a** The overall survival (OS) in the whole cohort; **b** the OS in the whole cohort according to the DS; **c** the OS in the whole cohort according to the ISS; **d** the OS in the whole cohort according to the RISS

A total of 638 eligible patients were enrolled in this study. The median age of the entire cohort was 60 years (interquartile range [IQR], 53–66 years). The median ages of the high and low NRI groups were 59 years (IQR, 53–66 years) and 62 years (IQR, 57–68 years), respectively. Three hundred and eighty-seven (60.2%) patients were men. There were no significant differences in sex or LDH, CRE, or Ca levels between the two groups. However, patients in the low NRI group were more likely to be older, have more advanced tumor stage, and have lower performance status and lower levels of β2-MG, CRP, and HGB and less likely to have undergone transplantation than those in the high NRI group; the differences were statistically significant. The median OS in the entire cohort was 58 months (5-year OS, 47.8%; 95% confidence interval [CI], 43.0–53.1; Fig. 1a). The baseline patient characteristics are presented in Table 1.

**Table 1:** Baseline characteristics of patients with NDMM (*n* = 638)

Variables	Total	NRI ≤ 89 (*n* = 211, %)	NRI > 89 (*n* = 427, %)	*p* value
Age (year), median (IQR)	60 (53–66)	62 (57–68)	59 (53–66)	
Age at diagnosis				0.045*
≤ 50	102 (16.0)	25 (11.8)	77 (18.0)	
> 50	536 (84.0)	186 (88.2)	350 (82.0)	
Sex				0.862
Male	387 (60.7)	82 (38.9)	169 (39.6)	
Female	251 (39.3)	129 (61.1)	258 (60.4)	
DS				< 0.001*
Stage I	38 (6.0)	10 (4.7)	28 (6.6)	
Stage II	119 (18.7)	19 (9.0)	100 (23.4)	
Stage III	481 (75.4)	182 (86.3)	299 (70.0)	
ISS				< 0.001*
Stage I	142 (22.3)	3 (1.4)	139 (32.6)	
Stage II	266 (41.7)	105 (49.8)	161 (37.7)	
Stage III	230 (36.1)	103 (48.8)	127 (29.7)	
R-ISS				< 0.001*
Stage I	108 (16.9)	1 (0.5)	107 (25.1)	
Stage II	433 (67.9)	165 (78.2)	268 (62.8)	
Stage III	97 (15.2)	45 (21.3)	52 (12.2)	
ECOG				0.002*
0–1	575 (90.1)	179 (84.8)	396 (92.7)	
≥ 2	63 (9.9)	32 (15.2)	31 (7.3)	
Transplant				0.029*
Yes	77 (12.1)	17 (8.1)	60 (14.1)	
No	561 (87.9)	194 (91.9)	367 (85.9)	
β2-MG (g/L)				< 0.001*
≤ 3.5	267 (41.8)	61 (28.9)	206 (48.2)	
> 3.5	371 (58.2)	150 (71.1)	221 (51.8)	
LDH (U/L)				0.052
≤ 250	520 (81.5)	163 (77.3)	357 (83.6)	
> 250	118 (18.5)	48 (22.7)	70 (16.4)	
CRP (mg/L)				< 0.001*
≤ 7.6	428 (67.1)	119 (56.4)	309 (72.4)	
> 7.6	210 (32.9)	92 (43.6)	118 (27.6)	
CRE (mmol/L)				0.176
≤ 177	574 (90.0)	185 (87.7)	389 (91.1)	
> 177	64 (10.0)	26 (12.3)	38 (8.9)	
HGB (g/L)				< 0.001*
≤ 120	481 (75.4)	193 (91.5)	288 (67.4)	
> 120	157 (24.6)	18 (8.5)	139 (32.6)	
Ca (mmol/L)				0.722
≤ 2.5	552 (86.5)	184 (87.2)	368 (86.2)	
> 2.5	86 (13.5)	27 (12.8)	59 (13.8)	

*NRI*, nutrition risk index; *DS*, Durie–Salmon staging system; *ISS*, international staging system; *R-ISS*, revised international staging system; *ECOG*, Eastern Cooperative Oncology Group; *β2-MG*, β2-microglobulin; *LDH*, lactate dehydrogenase; *CRP*, C-reactive protein; *CRE*, creatinine; *HGB*, hemoglobin; *Ca*, calcium

### Prognostic value of the DS staging system, ISS, R-ISS, and NRI for OS in MM patients

The DS staging system reflects tumor burden and does not predict the prognosis of MM; however, we evaluated its prognostic value in patients with NDMM in our study. The differences in OS between the groups were not significant (*p* = 0.26, Fig. 1b). Because the ISS and R-ISS staging systems have prognostic value in patients with MM, we explored their prognostic value in the entire cohort [5, 6]. The median OS of patients with ISS stages I, II, and III were 110 months, 53 months, and 45 months, respectively (*p* < 0.001, Fig. 1c). The median OS of patients with R-ISS stages I, II, and III were not reached, 57 months, and 25 months, respectively (*p* < 0.0001; Fig. 1d). Therefore, both the ISS and R-ISS staging systems predicted clinical outcome in this cohort.

We also assessed the prognostic ability of the NRI. The median OS of the low NRI group was 43 months (95% CI, 35–62 months), which was significantly shorter than that of the high NRI group (64 months; 95% CI, 56–93 months; *p* < 0.001; Fig. 1).

### Univariate and multivariate analyses of OS

In the univariate analysis of OS, older age (> 50 years; hazard ratio [HR], 1.838; 95% CI, 1.264–2.673; *p* = 0.001), poor performance status (Eastern Cooperative Oncology Group [ECOG] performance status score ≥ 2; HR, 2.096; 95% CI, 1.513–2.904; *p* < 0.001), high serum β2-MG (> 3.5 g/L; HR, 1.700; 95% CI, 1.322–2.186; *p* < 0.001), high serum LDH (HR, 2.069; 95% CI, 1.583–2.704; *p* < 0.001), high serum CRP (HR, 1.511; 95% CI, 1.185–1.926; *p* < 0.001), and high serum Ca (HR, 1.524; 95% CI, 1.089–2.135; *p* = 0.014) levels were significantly associated with worse OS in the entire cohort. Undergoing transplantation (HR, 0.368; 95% CI, 0.222–0.610; *p* < 0.001), high HGB level (HR, 0.565; 95% CI, 0.414–0.772; *p* < 0.001), and a high NRI (> 89; HR, 0.597; 95% CI, 0.468–0.760; *p* < 0.001) were significantly associated with better OS. However, in the multivariate analysis, only older age (HR, 1.509; 95% CI, 1.028–2.215; *p* = 0.008), ECOG performance status score ≥ 2 (HR, 2.096; 95% CI, 1.513–2.904; *p* < 0.001), undergoing transplantation (HR, 0.497; 95% CI, 0.297–0.833; *p* = 0.008), high serum LDH level (HR, 1.710; 95% CI, 1.297–2.253; *p* < 0.001), and a high NRI (HR, 0.758; 95% CI, 0.587–0.977; *p* = 0.033) were identified as independent prognostic factors in NDMM patients. Table 2 shows the results of the univariate and multivariate analyses of OS.

**Table 2:** Univariate and multivariate analyses of variables associated with overall survival

Variables	Univariate analysis		Multivariate analysis	
	HR (95% CI)	*p* value	HR (95% CI)	*p* value
Age (> 50 vs ≤ 50)	1.838 (1.264–2.673)	0.001	1.509 (1.028–2.215)	0.008*
Sex (female vs male)	1.059 (0.831–1.349)	0.643	-	
ECOG (≥ 2 vs 0–1)	2.096 (1.513–2.904)	< 0.001	1.791 (1.279–2.507)	< 0.001*
Transplant (yes vs no)	0.368 (0.222–0.610)	< 0.001	0.497 (0.297–0.833)	0.008*
β2-MG (> 3.5 vs ≤ 3.5 g/L)	1.700 (1.322–2.186)	< 0.001	1.194 (0.904–1.578)	0.212
LDH (> 250 vs ≤ 250 U/L)	2.069 (1.583–2.704)	< 0.001	1.710 (1.297–2.253)	< 0.001*
CRP (> 7.6 vs ≤ 7.6 mg/L)	1.511 (1.185–1.926)	< 0.001	1.219 (0.947–1.568)	0.124
CRE (> 177 vs ≤ 177 mmol/L)	1.175 (0.804–1.718)	0.405	-	
HGB (> 120 vs ≤ 120 g/L)	0.565 (0.414–0.772)	< 0.001	0.798 (0.566–1.124)	0.197
Ca (> 2.5 vs ≤ 2.5 mmol/L)	1.524 (1.089–2.135)	0.014	1.239 (0.878–1.749)	0.222
NRI (> 89 vs ≤ 89)	0.597 (0.468–0.760)	< 0.001	0.758 (0.587–0.977)	0.033*

**Fig. 2:**
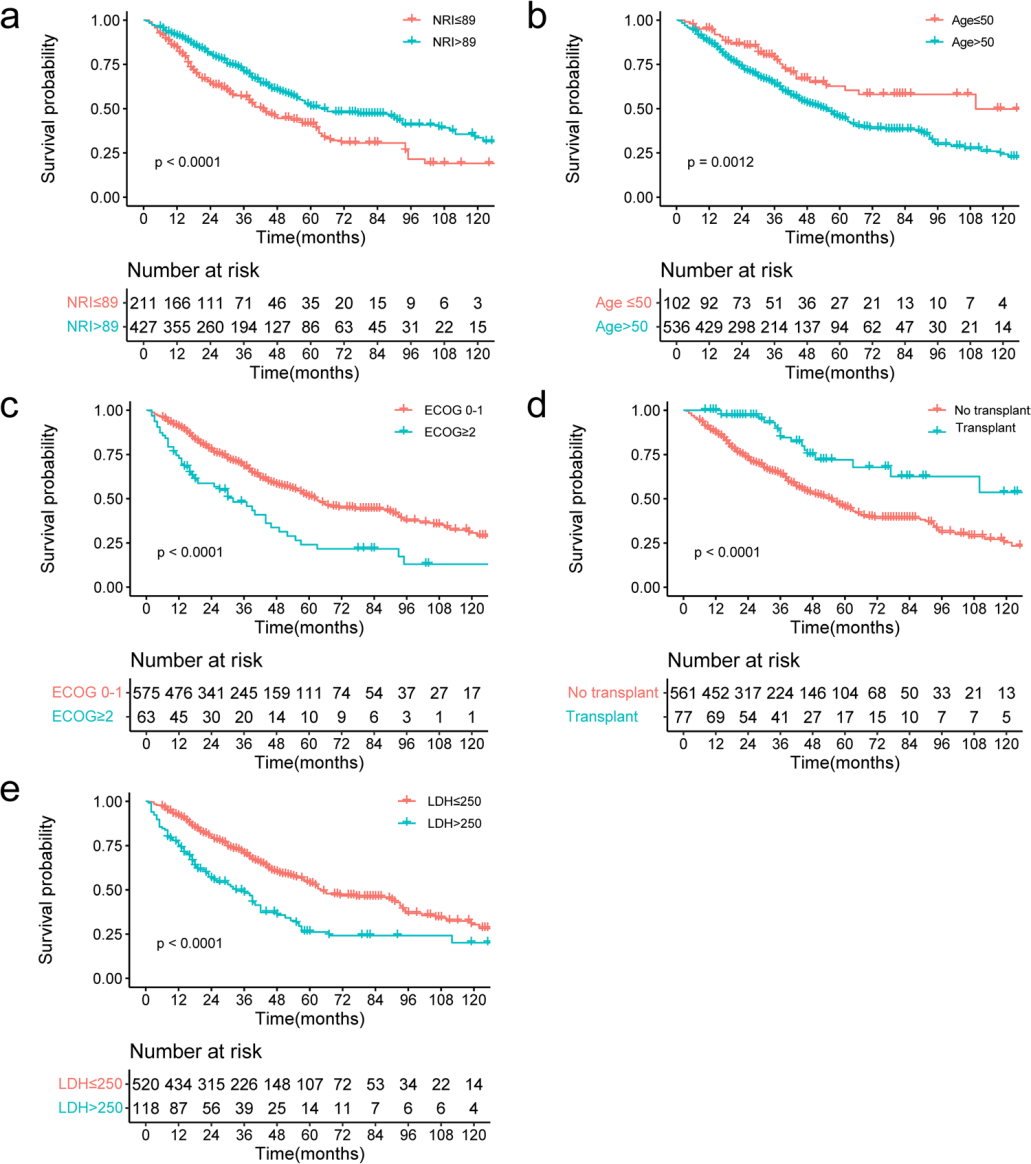
**a** The overall survival (OS) according to the NRI; **b** the OS in the whole cohort according to age; **c** the OS in the whole cohort according to ECOG; **d** the OS in the whole cohort according to transplant status; **e** the OS in the whole cohort according to LDH

The survival curves for the five independent prognostic factors are shown in Fig. 2. The median OS were 64 months (5-year OS, 51.0%; 95% CI, 45.0–57.7) and 43 months (5-year OS, 41.4%; 95% CI, 33.9–50.5; *p* < 0.001) in the high and low NRI groups, respectively.

## Discussion

Our study revealed that the NRI might be an independent prognostic factor of OS in patients with NDMM. Further, older age, ECOG performance status score ≥ 2, undergoing transplantation, and high serum LDH level were also independent prognostic factors for OS in NDMM patients. Patients in the high NRI group had a significantly better OS than those in the low NRI group. Consistent with the results of previous studies in other cancers, our results suggest that malnutrition, as assessed by the NRI, may be a predictor of poor survival in patients with NDMM [22–24].

The NRI is a comprehensive index based on albumin and weight and has been widely used in recent years to assess prognosis in cancer [25]. In our study, a low NRI was associated with a shorter OS. Based on the formula for calculating the NRI, a low NRI can indicate either low albumin levels or low weight. In our study, the proportion of patients with a poor performance status (ECOG performance status score ≥ 2) at diagnosis was significantly higher in the low NRI group than in the high NRI group. Patients in the low NRI group also had lower serum HGB levels than those in the high NRI group. This may be related to poor tolerance to chemotherapy and may, in part, explain the unfavorable survival outcomes. Moreover, only 8.1% of patients in the low NRI group received transplants. This implies that a low NRI is associated with poor tolerance to intensive chemotherapy. Better treatment tolerance allows for the prescription of higher doses of chemotherapy, thus improving survival. In addition, in our study, patients in the low NRI group were significantly more likely to be older and have high β2-MG levels, high CRP levels, and advanced disease than those in the high NRI group, which may also account for the worse survival outcomes in the low NRI group.

Albumin, a major protein in human serum, has been used to evaluate nutritional status, and low albumin levels have been shown to be related to poor prognosis in patients with various cancers [26–28]. In hematologic cancers, albumin has also been shown to be a strong predictor of prognosis and is one of the factors in the ISS [5]. Albumin can potentially carry a high amount of active antitumor compounds to the tumor site via albumin association [29]. Consequently, low albumin levels lead to a decreased probability of active antitumor compounds at the tumor site, which may be one of the reasons a low NRI was associated with poor prognosis. Low albumin levels can also cause high concentrations of certain drugs that cannot be converted into harmless substances, leading to toxicity over time; therefore, patients will suffer from severe adverse effects that can account for poor prognosis.

Obesity has been regarded as a risk factor for survival in many types of cancer, but some studies have shown that a low baseline BMI is also associated with poor survival in patients with cancer [30–32]. Unintended weight loss is commonly a sign of cancer and a negative predictor of survival. One retrospective study reported that a loss (> 5%) of premorbid weight before chemotherapy implied poor prognosis, independent of tumor stage, histology, and performance status [19]. Although multiple studies have examined the influence of obesity on the prognosis of various cancers, few studies have examined the influence of being underweight on the prognosis of patients with MM. A Korean study reported that a low baseline BMI was associated with poor prognosis in patients with MM. Western countries have a higher proportion of obese patients than Asian countries; therefore, being underweight may play a more critical role than being obese in predicting the prognosis of Asian patients with MM.

Our study has some limitations. First, it was a retrospective study. Second, the study was performed in a single center and not in multiple centers. Third, we did not explore the potential mechanism by which the NRI affects the prognosis of NDMM. Therefore, further studies with larger sample sizes are required to verify the results.

In conclusion, we found that the NRI might be a prognostic factor for NDMM. The NRI is easy to acquire and could be a simple and useful implement for forecasting the prognosis of NDMM. Further multicenter investigations are needed to confirm these findings.

## Data Availability

The data supporting our study will be available by contacting the corresponding authors for reasonable reasons.
